# Sustainable Stabilizer-Free Nanoparticle Formulations of Valsartan Using Eudragit^®^ RLPO

**DOI:** 10.3390/ijms222313069

**Published:** 2021-12-02

**Authors:** Eszter Hajba-Horváth, Andrea Fodor-Kardos, Nishant Shah, Matthias G. Wacker, Tivadar Feczkó

**Affiliations:** 1 Faculty of Engineering, Research Institute of Biomolecular and Chemical Engineering, University of Pannonia, Egyetem u. 10, H-8200 Veszprém, Hungary; hajba-horvath@mukki.richem.hu (E.H.-H.); kardos@mukki.richem.hu (A.F.-K.); 2 Research Centre for Natural Sciences, Institute of Materials and Environmental Chemistry, Magyar Tudósok Körútja 2, H-1117 Budapest, Hungary; 3 College of Pharmacy, University of Michigan, 500 S State Street, Ann Arbor, MI 48109, USA; shahn9211@gmail.com; 4 Department of Pharmacy, National University of Singapore, 4 Science Drive 2, Singapore 117544, Singapore

**Keywords:** nanoparticles for drug delivery, valsartan, Eudragit^®^, emulsion-solvent evaporation, drug release, in silico modeling

## Abstract

The bioavailability of the antihypertensive drug valsartan can be enhanced by various microencapsulation methods. In the present investigation, valsartan-loaded polymeric nanoparticles were manufactured from Eudragit^®^ RLPO using an emulsion–solvent evaporation method. Polyvinyl alcohol (PVA) was found to be a suitable stabilizer for the nanoparticles, resulting in a monodisperse colloid system ranging in size between 148 nm and 162 nm. Additionally, a high encapsulation efficiency (96.4%) was observed. However, due to the quaternary ammonium groups of Eudragit^®^ RLPO, the stabilization of the dispersion could be achieved in the absence of PVA as well. The nanoparticles were reduced in size (by 22%) and exhibited similar encapsulation efficiencies (96.4%). This more cost-effective and sustainable production method reduces the use of excipients and their expected emission into the environment. The drug release from valsartan-loaded nanoparticles was evaluated in a two-stage biorelevant dissolution set-up, leading to the rapid dissolution of valsartan in a simulated intestinal medium. In silico simulations using a model validated previously indicate a potential dose reduction of 60–70% compared to existing drug products. This further reduces the expected emission of the ecotoxic compound into the environment.

## 1. Introduction

Bioavailability is a key requirement for the application of bioactive compounds in pharmaceutical drug products [1]. Therapeutic efficacy widely depends on this important characteristic, describing the percentage of the drug that appears in blood circulation. Novel formulation strategies often lead to improved bioavailability and allow dose reduction while achieving similar therapeutic effects in patients [2]. Additionally, they contribute to more sustainable use of drug substances by reducing drug emissions released into the environment [3].

Many efforts have been made to enhance the bioavailability of therapeutic agents that exhibit poor aqueous solubility. One common strategy is the development of nanoformulations for drug delivery [4,5]. These include polymeric nanoparticles, solid lipid nanoparticles, liposomes, nanostructured lipid carriers, nanoniosomes, and nanoemulsions [6]. Eudragit^®^ is a trademark of synthetic poly(meth)acrylate polymers with varying monomer compositions. Common monomers include methacrylic acid, methacrylic acid esters, and dimethylaminoethyl methacrylate. Their pH-dependent water solubility enables the modulation of the drug release in different segments of the human gastrointestinal tract. Therefore, they are widely applied in the production of microspheres, microparticles, and nanoparticles [7].

Valsartan, an antagonist of the selective angiotensin II type 1 receptor [8] is an antihypertensive drug. It exhibits a peroral bioavailability of only 23–25% [9]. In recent years, due to the excessive use of valsartan in the treatment of cardiovascular diseases, significant amounts were found in the surface waters of many countries [10,11]. Consequently, the drug represents an optimal candidate for the development of more sustainable drug formulations with improved bioavailability.

In previous investigations, mesoporous silica nanoparticles were used to enhance the oral bioavailability and antihypertensive activity of valsartan [12]. They were functionalized with aminopropyl groups and coated with the pH-sensitive polymer Eudragit^®^ L100-55. The solubility of the drug was improved by stabilizing the amorphous state of the drug, leading to more rapid dissolution. Expectedly, a pH-dependent sustained-release behavior was observed. After preparation, the valsartan-loaded nanoparticles were characterized in vitro and in vivo [13].

Recently, we reported the preparation of valsartan-loaded particles comprising ethylcellulose and poly(methyl methacrylate). The polymer composition and type were used to control release [14]. To date, Eudragit^®^ RLPO, a copolymer of ethyl acrylate and methyl methacrylate, has been widely used for preparing nanoparticles, mucoadhesive tablets, patches, and solid dispersions [15]. The pore structure enables the preparation of sustained-release drug delivery systems [16]. Gandhi and coworkers used Eudragit^®^ RLPO for the encapsulation of acyclovir using the nanoprecipitation method. Pluronic^®^ F68 served as a stabilizing agent to reduce the agglomeration of the particles. The drug was kept in the amorphous state and was homogeneously dispersed in the polymer matrix. The in vitro release of acyclovir was effectively sustained for 24 h with a total release ranging from 71–93%.

The present work utilizes the emulsifying properties of Eudragit^®^ RLPO to create a highly stable drug delivery system in the absence of Pluronic F68 or polyvinyl alcohol (PVA). Although highly effective with regard to their stabilizing properties, many of these colloid stabilizers exhibit a certain degree of ecotoxicity. For example, PVA was reported to be poorly degradable in a marine environment [17]. Therefore, by reducing or replacing these inactive ingredients in the formulation, a reduction in the emission of ecotoxins is expected. The polymer Eudragit^®^ RLPO has a positive surface charge and can more easily be extracted from wastewater [18,19]. Therefore, the current formulation design contributes to the more sustainable use of excipients in the pharmaceutical industry.

Among other important characteristics, we examined the particle size, size distribution, and encapsulation efficiency of the novel formulations. Particle size is an important characteristic with regard to dissolution rate and bioavailability. Nanoparticles of the smallest size, a narrow size distribution, and the highest encapsulation efficiency were selected for further investigations. Additionally, the drug-loaded particles were compared to colloids manufactured in the absence of the drug to elucidate the influence of the drug substance on the preparation process. To understand the surface-active behavior of Eudragit^®^ RLPO, the interfacial tension (IFT) of a solution of the polymer was measured in presence of 1% of PVA. Polymer solutions in the presence and absence of the drug were compared. After optimizing the formulation characteristics, bioavailability was estimated using a biorelevant dissolution set-up [20] together with in silico modeling [21]. Conditions in the human gastrointestinal tract were simulated in vitro using fasted-state simulated gastric fluid (FaSSGF) and fasted-state simulated intestinal fluid (FaSSIF).

## 2. Results and Discussion

Currently, a broad array of formulation strategies is aiming at the development of more sustainable drug products with high therapeutic efficacy. Here, we describe the synthesis of valsartan-loaded nanoparticles using the sustained-release polymer Eudragit^®^ RLPO. The selected formulation characteristics were optimized with regard to particle size, size distribution, and encapsulation efficiency. Further, we prepared the nanoparticles in the presence and absence of the colloid stabilizer PVA. The final formulation design provides a stabilizer-free dispersion with optimal biopharmaceutical properties as indicated by the biorelevant two-stage in vitro dissolution test together with in silico modeling.

### 2.1. Physicochemical Characteristics of Eudragit^®^ RLPO Nanoparticles

The average hydrodynamic diameter of valsartan-loaded Eudragit^®^ RLPO nanoparticles ranged from 134.0 nm to 169.7 nm, with a polydispersity index (PDI) between 0.090 and 0.158. This indicates a monodisperse size distribution. Encapsulation efficiencies between 77.5% and 96.4% were determined. The preparation processes resulted in process yields ranging from 59.2–86.1% (Table S1 in Supplementary Materials). In preliminary experiments, we identified critical process variables, such as the emulsifier and polymer concentrations. The volume of the organic and the water phase, as well as the drug concentration, were kept constant.

**Figure 1:**
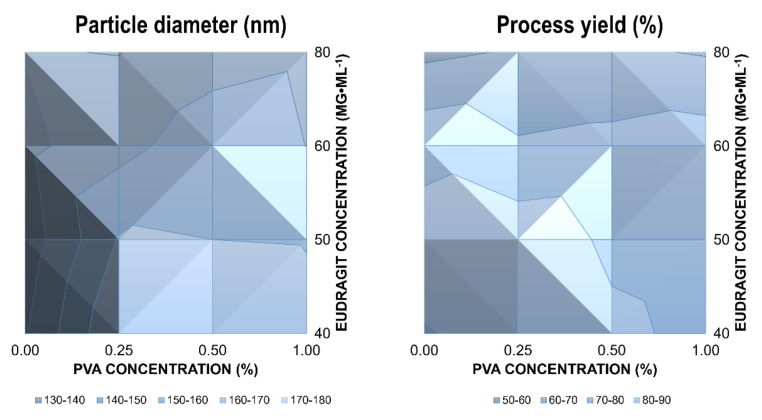
Influence of PVA (%) and Eudragit^®^ concentration (mg·ml^−1^) on particle diameter (nm, (**left**)) and process yield (%, (**right**)). A change in color depth indicates a change in the respective parameter, as outlined in the legend.

The mean diameter of the nanoparticles was not affected by the PVA concentration over a concentration range from 0.25–1.0% (Figure 1, left). On the contrary, increasing Eudragit^®^ concentrations from 40–80 mg·ml^−1^ resulted in a significant decrease in size. This indicates a potential emulsifying effect of Eudragit^®^ RLPO. Consequently, nanoparticles were synthesized in the absence of PVA to elucidate the effects on the physical stability of the dispersion. The particle size of drug-loaded nanoparticles was much smaller in the absence of PVA (Figure 1, left). Additionally, the Eudragit^®^ RLPO concentration had no significant influence on particle size, indicating robust self-assembly of the colloids. Chernyseva et al. previously reported emulsifying properties of Eudragit^®^ RLPO [22]. They examined the effect of the hydrophobic polymer on emulsion droplet size and nanoparticle size using the emulsification–solvent evaporation method. In our investigations, increasing PVA concentrations were accompanied by a slight reduction in encapsulation efficiency (Table 1). The effect was even more pronounced for the polymer concentration.

The process yield indicates similar trends (Figure 1). At higher concentrations of polymer and stabilizer, the process yield was reduced. However, while the variations in the process yield were not significant between different PVA concentrations, an increase in the Eudragit^®^ concentration resulted in a significant reduction of the process yield (ANOVA, *p* < 0.05). This can be explained by the stronger emulsifying properties of Eudragit^®^ RLPO.

**Figure 2:**
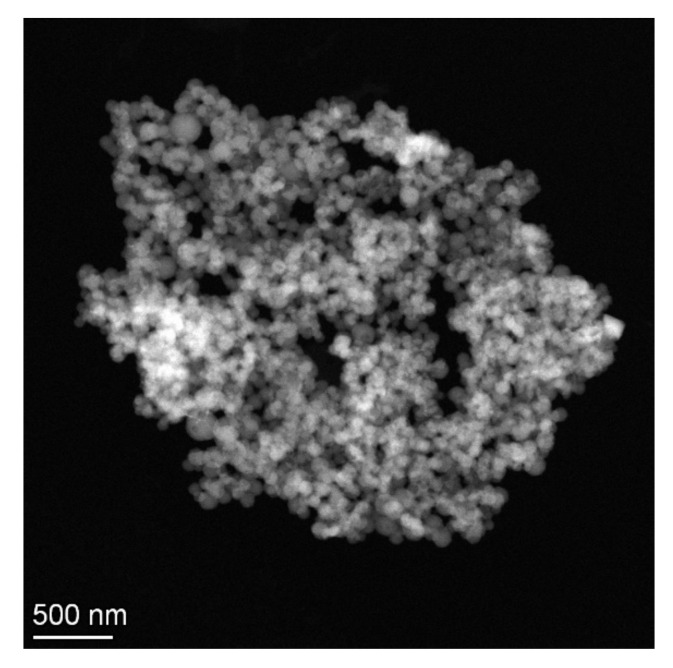
S/TEM images of valsartan-loaded Eudragit^®^ RLPO nanoparticles.

These experiments were conducted as part of the formulation screening, and replicates were made for the optimized formulation. A total of six independent samples were prepared at the highest Eudragit^®^ RLPO concentration in the absence and presence of PVA (1% m/v). The average particle size in the absence of PVA was 133.9 ± 2.5 nm, while a diameter of 152.9 ± 2.6 nm was determined in the presence of the stabilizer. A more detailed summary of formulation parameters and the results is provided in the Supplementary Materials (Table S2). Particle size was confirmed by scanning transmission electron microscopy (S/TEM). Monodisperse size distribution was observed (Figure 2).

Both parameters, size and encapsulation efficiency, were modified by altering the process parameters using Eudragit^®^ RLPO in a self-stabilizing dispersion. The quaternary ammonium groups are the most likely explanation for the emulsifying effect [23]. This led to a stabilizer-free formulation with high encapsulation and small particle size. To use this versatile manufacturing process as a platform technology for other compounds, a better understanding of the impact of the drug molecule on the physicochemical characteristics was required.

### 2.2. Particle Size of Blank Eudragit^®^ RLPO Nanoparticles

To explore the impact of valsartan on the properties of the nanoparticles and to study the emulsifying effect of the polymer, nanoparticles were prepared in absence of the drug. Furthermore, we investigated the emulsifying effect of Eudragit^®^ RLPO in more detail, comparing the preparation in water and at three different PVA concentrations. As outlined in Table 2, the particle size of the blank nanoparticles was much smaller, and the PDI increased substantially compared to the valsartan-loaded nanoparticles. The larger diameter in the presence of the drug can be explained by the higher solid content of the dispersion. The lowered PDI could indicate an interaction between the encapsulating polymer and the drug; however, it may also be due to the overall lowered scattering intensity observed at smaller particle sizes. Commonly, larger particles exhibit increased scattering intensity. This changes the ratio between the scatter signal of the major particle fraction and small-particulate impurities with an impact on the PDI.

The concentration of the emulsifier did not affect the examined parameters significantly. However, for blank nanoparticles prepared in the absence of the emulsifier, an increase in particle size was observed. Chernyseva and coworkers [22] reported a detectable but not significant increase in the emulsifying properties of the examined Eudragit^®^ polymers in the presence of PVA in the aqueous phase of the emulsions. On the contrary, we were not able to detect any correlation between PVA concentration and particle size. In the referred work, the researchers studied polymer nanoparticles in the absence of the drug molecule.

The zeta potential (Table 2) indicates charge stabilization of the Eudragit^®^ RLPO nanoparticles. Our results were very similar to the ones reported by Chernysheva et al. [22]. The authors measured zeta potentials of +42 ± 3 mV for Eudragit^®^ RS particles and +53 ± 1 mV for Eudragit^®^ RL particles. The high stability of the suspensions is likely related to the presence of quaternary ammonium groups. Koch et al. reported enhanced electrochemical stability for polymers comprising quaternary ammonium derivatives [24].

### 2.3. Interfacial Tension Measurements

IFT measurements were required to further elucidate the driving forces involved in particle self-assembly. The data are summarized in Table 3.

The density and surface tension of the two solutions did not differ significantly. The IFT was found to be substantially lower in 1% PVA solution as compared to purified water. This can be explained by the emulsifying properties of PVA.

Chernyseva et al. measured an IFT of 3 ± 1 mN/m for Eudragit^®^ RL and 4 ± 1 mN/m for Eudragit^®^ RS dissolved in DCM and emulsified in water [22]. Despite the differences in polymer type, concentration, and solvent, these reported values were well aligned with our findings (Table 3). A comparison of the two solutions with and without valsartan showed a lowered IFT in water and a higher IFT in 1% PVA.

The decrease in the IFT observed for valsartan-loaded nanoparticles was expected to result in smaller particle sizes. At concentrations of 80 and 160 mg of Eudragit^®^ RLPO, average particle sizes of 139.1 nm and 120.4 nm were determined, respectively. For comparison, the average diameter of the blank nanoparticles was 74.8 nm (80 mg of polymer) and 106.3 nm (160 mg of polymer), respectively. This was an unexpected finding and cannot be explained with IFT alone. Agglomeration effects could have contributed to a more rapid particle growth in the presence of the drug. An inverse relationship was found when PVA was present. Under these conditions, IFT and particle size increased when valsartan was added during preparation. An amount of 80 mg of Eudragit^®^ RLPO resulted in particle sizes of 80.8 nm in the absence of the drug and 162.3 nm in its presence. When using 160 mg of the polymer, particle sizes of 99.6 (without valsartan) and 148.4 nm (with valsartan) were measured. At this concentration, PVA may act as a steric colloid stabilizer. Still, Eudragit^®^ RLPO can self-assemble nanoparticles.

### 2.4. In Vitro Drug Release

A biorelevant drug release study was carried out with the valsartan-loaded Eudragit^®^ RLPO nanoparticles at gastric and intestinal pH using a two-stage dissolution set-up.

**Figure 3:**
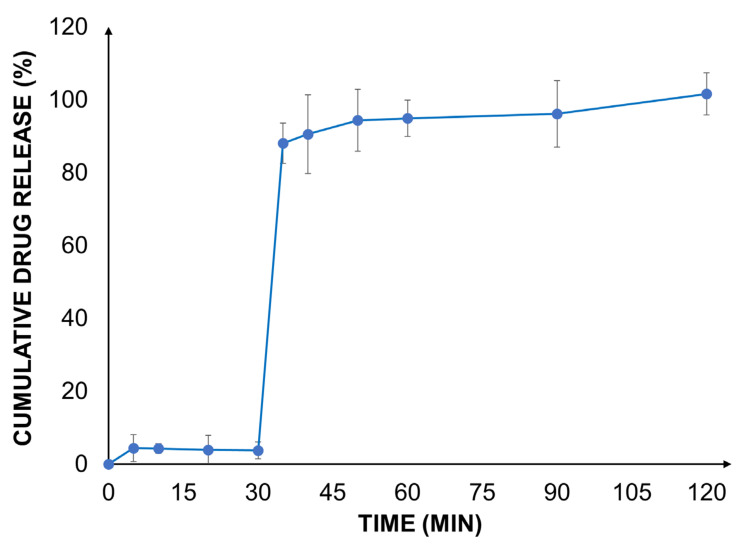
In vitro release profile of valsartan from Eudragit^®^ RLPO–valsartan nanoparticles in FaSSGF for 30 min and in FaSSIF for a further 90 min according to the two-stage biorelevant pH-shift dissolution test.

As expected, in the gastric medium, the dissolution and release of the drug were very low due to the poor aqueous solubility of the active ingredient at an acidic pH (Figure 3). This behavior was likely influenced by the presence of the poorly soluble polymer, further delaying the release of the drug. Similar observations have been made previously [25].

In the biorelevant two-stage dissolution setup, a significant increase in the drug release rate was observed after the addition of FaSSIF to each vessel. At the end of the experiment, a complete release of the encapsulated drug from the nanoparticles was found. The outcome was well aligned with earlier findings [25]. This can be explained by an increased solubility of valsartan as well as the improved water permeability and reduced swelling of Eudragit^®^ RLPO. These effects contribute to an improved drug load and accelerate the release of valsartan. The impact of the improved release behavior on the biopharmaceutical characteristics of the formulation was estimated by in silico modeling.

### 2.5. Modeling of Human Pharmacokinetics

**Figure 4:**
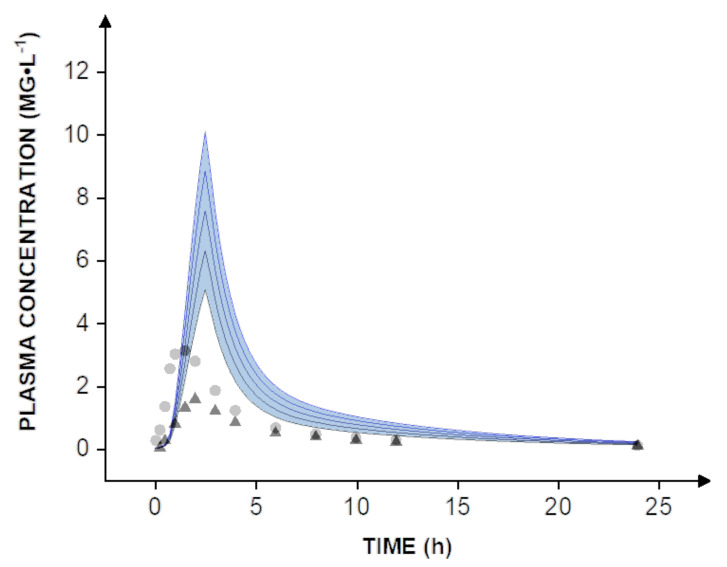
Simulation of human pharmacokinetics following single-dose administration of 40, 50, 60, 70, and 80 mg of valsartan nanoparticles (blue lines) and, for comparison, mean plasma concentrations observed after the administration of a solution (gray circles) and a capsule formulation (dark gray triangles), comprising 80 mg of valsartan to 12 healthy volunteers.

The human pharmacokinetics of valsartan following intravenous and peroral administration of the drug was reported previously [8]. The clinical data include peroral administration of a capsule formulation and a drug solution at a dose of 80 mg, as well as pharmacokinetics after intravenous injection of 20 mg of valsartan. The optimized drug-loaded Eudragit^®^ nanoparticles were tested in vitro at a dose of 40 mg. To estimate pharmacokinetics and enable a direct comparison to human clinical data, dose scaling from 40-80 mg was carried out in silico (Figure 4, blue lines).

Expectedly, even the administration of a solution of valsartan did not compare to the nanoparticle formulation, which reaches a bioavailability of almost 100%. The increase is most apparent when comparing the simulation to human clinical data obtained with the capsule formulation. On average, the solution resulted in a peroral bioavailability of 39%, while the capsule formulation reached approximately 23% [8]. In this context, it must be considered that all simulations are widely based on in vitro data including the dissolution rate and permeability. Therefore, further in vivo studies would be necessary to determine the human bioavailability of valsartan. However, the simulations suggest that a dose reduction of at least 60–70% could be achieved by using a nanotechnology-based formulation design.

## 3. Materials and Methods

### 3.1. Modeling of Human Pharmacokinetics

Valsartan was kindly provided by EGIS Pharmaceuticals PLC (Budapest, Hungary). Eudragit^®^ RLPO was also a kind gift from Evonik Röhm GmbH (Darmstadt, Germany), PVA (87–90% hydrolyzed, M_W_ = 30,000–70,000) was purchased from Sigma-Aldrich (St. Louis, MO, USA). Dichloromethane (DCM) (CARLO ERBA Reagents S.A.S., Val de Reuil Cedex, France) and ethyl acetate (EtOAc) were purchased from Scharlab Hungary Ltd. (Debrecen, Hungary). FaSSIF/FaSSGF powder and FaSSGF buffer were purchased from Biorelevant.com Ltd. (London, UK). NaCl, NaOH, NaH_2_PO_4_• H_2_O were purchased from Reanal Ltd. (Budapest, Hungary).

### 3.2. Formulation of Valsartan-Loaded Eudragit^®^ RLPO Nanoparticles

A single-emulsion-solvent evaporation process was used for the synthesis of the drug-loaded nanoparticles. To evaluate the effect of experimental parameters (polymer, drug, and emulsifier concentration) on the particle size, size distribution, yield, and encapsulation efficiency of the obtained nanocomposites, a series of samples were prepared by the modification of process parameters. An oil–water (o/w) emulsion system comprising 80–160 mg of Eudragit^®^ RLPO was dissolved in 2 mL of DCM. For the dissolution of the active ingredient, a volume of 1 mL of ethyl acetate (EtOAc) was selected. The dissolved amount of valsartan was 40 mg for each batch. The polymer and drug solutions (total volume of 3 mL of organic phase) were emulsified with a Sonics VCX130 sonicator (Sonics & Materials, Inc., Newtown, CT, USA) in 6 mL of water in the absence or presence of 0.25%, 0.5%, and 1% PVA emulsifier for 30 s. After the emulsification process, solvent removal from the system was carried out with evaporation overnight at room temperature and 1 bar using an IKA RCT magnetic stirrer (IKA-Werke, Staufen, Germany).

### 3.3. Formulation of Valsartan-Loaded Eudragit^®^ RLPO Nanoparticles

To study the emulsifying effect of the Eudragit^®^ RLPO polymer, nanoparticles without the drug were also prepared with the same method as was used in the case of the valsartan-loaded particles (see Section 3.2). The amount of polymer used was 80 mg or 160 mg in the blank particles. These values correspond to the minimum and maximum amount of polymer among the tested polymer concentrations in the valsartan-loaded nanoparticles. The blank particles with both polymer contents were prepared at each applied PVA concentration (0.25%, 0.5%, and 1%) and without any emulsifier. A total of 2 mL of DCM was used as solvent of the polymer, then 1 mL of EtOAc was added to the solution. The nanoparticles were also obtained with a single-emulsion–solvent evaporation process as described in Section 3.2.

### 3.4. Characterization of Nanoparticles

#### 3.4.1. Particle Size and Morphology

The dynamic light scattering (DLS) method was used for the determination of average particle size and size distribution. The measurements were carried out using nanoparticle suspensions after preparation without further dilution. The viscosity of each sample was measured using an SV-10 vibrational viscometer (A&D, Tokyo, Japan). Five size measurements were carried out for each sample with a Malvern Zetasizer ZS (Malvern Instruments, Malvern, UK) equipped with a backscatter detector detecting at an angle of 173° in a single-use cuvette. The particles were characterized for their intensity mean diameter and PDI at a temperature of 25 °C. The nanoparticle morphology was investigated using an FEI Talos F200XG2 scanning transmission electron microscope (Thermo Fischer Scientific, Waltham, MA, USA) operated at 200 keV.

#### 3.4.2. Yield

The valsartan-loaded nanosuspension samples were microcentrifuged at 15,000 rpm for 30–60 min with a Hermle Z216 MK Microcentrifuge (Hermle AG, Gosheim, Germany). After discarding the supernatant, the pellets were dried at 40 °C for 48 h until a constant weight was reached. The yield was calculated based on the particle mass in the entire sample, and the theoretically weighed combined mass of the polymer and drug.

#### 3.4.3. Drug Encapsulation Efficiency

The valsartan entrapped in the nanoparticles was determined by a spectrophotometric method using a UV-1800 UV–vis spectrophotometer (Shimadzu Corporation, Kyoto, Japan). The non-encapsulated drug was measured in the supernatant after microcentrifugation with a Hermle Z216 MK Microcentrifuge (Hermle AG, Gosheim, Germany) of the nanosuspensions. Calibration was prepared at the applied concentrations of the emulsifier with a detectable linear calibration range from 2.5–40 µg/mL. In the case of samples that were formulated without PVA emulsifier, a phosphate-buffered solution at pH 6.8 was used for calibration in the same concentration range. The absorbance of the diluted sample solutions was measured at 250 nm.

#### 3.4.4. Zeta Potential

The zeta potential of prepared nanosuspensions was measured by a Malvern Zetasizer ZS (Malvern Instruments, Malvern, UK) at 25 °C. Before zeta potential measurements, the samples were prepared according to the following procedure: 1 mL or 2 mL of the suspensions (depending on the particle size) was centrifuged at 80,000 rpm for 30 min with a Hermle Z216 MK Microcentrifuge (Hermle AG, Gosheim, Germany). After centrifugation, the supernatant was discarded, and the pellet was washed with 1 mL of MilliQ water to remove the residual PVA from the surface of the particles. This step of the procedure was necessary because the presence of the emulsifier interferes with the zeta potential measurement. In the case of the blank samples prepared without an emulsifying agent, centrifugation was therefore omitted, and 1 mL of these samples was directly measured. Five parallel measurements were carried out for each sample according to the Smoluchowski model using a zeta dip cell. After the measurements, the cell was cleaned with ultrasonication (Sonics VCX130 sonicator, Sonics & Materials Inc., Newtown, CT, USA) for 30 s at 20%.

### 3.5. Interfacial Tension Measurements

IFT measurements were carried out to examine the assumed emulsifying effect of Eudragit^®^ RLPO polymer and the potential function of drug in the nanoparticle formation without an emulsifier. The polymer was dissolved in a dichloromethane—ethyl acetate solvent mixture, and the solution of the polymer and the drug together was also prepared with the same solvents. Both sample solutions contained Eudragit^®^ RLPO and valsartan in the highest concentration, which was applied during the preparation of the series of emulsion samples. Their volume was chosen according to the sample requirements of the IFT measurements. A total of 1.12 g polymer was dissolved in 14 mL of DCM, then 7 mL of EtOAc was added to the solution. Another solution contained the same amount of Eudragit^®^ RLPO and 280 mg of valsartan in the same type and volume of solvents.

The density of the prepared solutions was determined with the help of a series of weight measurements of solutions with a volume of 1 mL. The average measured values were considered in the IFT measurements. The IFT of polymer solution with and without valsartan was determined in MilliQ water and 1% PVA with an FTA 1000 B tensiometer (First Ten Angstroms, Portsmouth, NH, USA) using the pendant drop method evaluated with the FTA32 Video 2.1 software. As a first step, the surface tension of solutions was measured to determine the IFT between the examined liquid surfaces. Based on these data, it was possible to carry out the IFT measurements with the appropriate software settings. The video files recorded during the IFT measurements were analyzed. All results were evaluated in Excel (Microsoft, Redmont, WA, USA).

### 3.6. In Vitro Drug Release Studies

The release of valsartan from the nanoparticles was determined using a two-stage biorelevant pH-shift dissolution test method. The nanoparticles were prepared in the presence of 1% of PVA. During this procedure, a volume of 2 L of FaSSGF was prepared using FaSSIF–FaSSGF biorelevant powder and FaSSGF buffer concentrate. Additionally, 1.5 L of FaSSIF double concentrate was prepared with this powder and FaSSIF phosphate buffer, for which the surfactant concentration and buffer strength were doubled compared to FaSSIF. Six parallel dissolution vessels were equilibrated to 37 °C ± 0.5 and stirred at 75 rpm with a magnetic stirrer containing 250 mL of FaSSGF in each dissolution vessel. The samples were ingested in the dissolution medium in the form of centrifuged and redispersed suspensions. A total of 6 mL of the nanosuspensions were ultracentrifuged at 40,000 rpm for 20 min with an Optima Max Ultracentrifuge (Beckman Coulter, Indianapolis, IN, USA); then, the nanoparticles were redispersed in 1 mL of FaSSGF. In addition, drug encapsulation efficiency was determined according to Section 3.4.3 to determine the drug load of nanosuspensions. The dissolution test was run in this medium with sampling points at 5, 10, 20, and 30 min. The samples were filtered through a filter with a cut-off of 0.05 µm. After each sample was taken, the volume of the sample was replaced with pre-warmed FaSSGF. After 30 min, 250 mL of pre-warmed FaSSIF double concentrate was added to each dissolution vessels. Samples were collected from this mixed media 35, 40, 50, 60, 90, and 120 min after the beginning of the test. The samples were also filtered using a filter with a cut-off of 0.05 µm. The volume of the removed samples was replaced with a pre-heated 1:1 mixture of FaSSGF and FaSSIF double concentrate. The first 2–3 mL was discarded before collecting the samples for analysis in the case of both media. The samples were analyzed by a spectrophotometric method using a Shimadzu UV-1800 UV–vis spectrophotometer (Cole-Parmer, Vernon Hills, IL, USA). The calibration curve was prepared with a 1:1 mixture of FaSSGF and FaSSIF double concentrate with a detectable linear calibration range of 2.5–40 µg/mL. The composition of FaSSGF biorelevant dissolution media and the FaSSIF buffer, which was used to prepare the FaSSIF double concentrate biorelevant media, is presented in Table 4.

NaOH (aq) was added to adjust the pH of the FaSSIF buffer to 7.5. The FaSSIF double concentrate dissolution media was prepared by adding 1.5 L of FaSSIF buffer to 8.96 g of FaSSIF/FaSSGF biorelevant powder.

### 3.7. Modeling the Pharmacokinetics of Nanoparticles

To simulate human pharmacokinetics of the nanoparticles, a physiologically based biopharmaceutics model (PBBM) was utilized. The model simulations were carried out in Stella Architect^®^ (isee systems, Lebanon, PA, USA). The transfer model provides a combined in vitro release experiment mimicking drug release in the stomach and the upper intestine. Hence, only one in vitro release profile was implemented to describe the dissolution step. Permeation into blood circulation was modeled as reported previously [3,26,27]. In brief, the released drug enters a compartment representing the unstirred water layer before permeation into blood circulation occurs. Important model parameters are summarized in Table 5.

A linear extrapolation between the measured time points enabled the calculation of the slope of the observed dissolution profile at every simulated time point (every 18 s). This converted in vitro profile was used to simulate the transport of the drug into a compartment representing the unstirred water layer. The mucin layer has a smaller surface area and is characterized by poor hydrodynamics. From there, the drug slowly diffuses to the gut wall from where it is absorbed into blood circulation. The distribution and elimination were calculated using the circulation and recirculation rate constant (k_12_, k_21_), the elimination rate constant (k_e_), and the volume of distribution (V_D_) of valsartan obtained by pharmacokinetic analysis of human clinical data reported previously [8]. After the extraction of the plasma concentration-time profile, Monolix 2020 Suite (Lixoft, Antony, France) was used to fit the data with a standard two-compartment model.

## 4. Conclusions

Valsartan-loaded Eudragit^®^ RLPO polymeric nanoparticles were synthesized using the single-emulsion–solvent evaporation method. Originally, the nanoparticles were prepared using PVA as a stabilizer. As part of our investigation, we successfully demonstrated the preparation without PVA, resulting in smaller particle sizes (134–141 nm as compared to 148–162 nm). The drug encapsulation efficiency was similarly high without the stabilizing agent (82–96%). In this context, we took advantage of the excellent emulsifying properties of Eudragit^®^ RLPO. This was confirmed by the IFT measurements as well. The particle system led to optimal release properties in FaSSIF. Based on the in silico simulations, we estimated a dose reduction of 60–70% compared to the capsule formulation.

## Figures and Tables

**Table 1:** Encapsulation efficiency of valsartan-loaded nanoparticles as a function of polymer and emulsifier concentration.

Eudragit^®^ RLPO Concentration in DCM (mg/mL)	Encapsulation Efficiency (% m/m)			
40.0	96.4	96.4	95.2	94.2
50.0	96.2	91.8	92.0	92.3
60.0	92.3	91.6	87.5	89.8
80.0	82.4	84.6	80.7	77.5
	0	0.25	0.5	1
	PVA concentration (% m/v)			

**Table 2:** Experimental parameters of blank Eudragit^®^ RLPO nanoparticles prepared by single-emulsion–solvent evaporation method and the measured average particle sizes, polydispersity indices, and zeta potential values.

Sample ID	Eudragit^®^ RLPO Concentration in DCM (mg/mL)	PVA Concentration (% m/v)	Mean Size by Intensity (nm)	PDI	Zeta Potential (mV)
EUV43	40.0	0.0	74.8	0.268	+55.1
EUV44	80.0	0.0	106.3	0.298	+44.0
EUV49	40.0	0.25	80.2	0.348	+66.8
EUV50	80.0	0.25	115.3	0.274	+60.3
EUV47	40.0	0.5	81.2	0.323	+60.7
EUV48	80.0	0.5	94.5	0.270	+65.6
EUV45	40.0	1.0	80.8	0.311	+63.0
EUV46	80.0	1.0	99.6	0.283	+64.7

**Table 3:** Density, surface tension, and IFT of the polymer in MilliQ water and in 1% PVA solution in presence and absence of valsartan.

Solution	Density (g/cm^3^)	Surface Tension (mN/m)	IFT in Purified Water (mN/m)	IFT in 1% PVA (mN/m)
Eudragit^®^ RLPO without valsartan	1.198	27.94	2.94	0.46
Eudragit^®^ RLPO with valsartan	1.151	27.09	1.89	0.71

**Table 4:** Composition of FaSSGF biorelevant dissolution media and FaSSIF buffer.

Compound	FaSSGF (2L)	FaSSIF Buffer (1.5 L)
Biorelevant powder (g)	0.120	
FaSSGF buffer concentrate (ml)	70.35	
Purified water (g)	1933.42	~1.9 L
NaOH (g)		~1.68
NaH_2_PO_4_ H_2_O (g)		15.82
NaCl (g)		24.74

**Table 5:** Modeling parameters used for the simulation of human pharmacokinetics.

Parameter	Value	Reference
Permeability (cm × s^−1^)	6.6 × 10^−6^	[28]
Area of the mucin layer [cm^2^]	2286 cm^2^	[3,21]
Intestinal surface area [cm^2^]	23,323 cm^2^	[3,21]
k_12_ (h^−1^)	0.307	[8]
k_21_ (h^−1^)	0.216	[8]
k_e_ (h^−1^)	0.425	[8]
V_D_ [mL]	4689.67	[8]

## Data Availability

Not applicable.

## Supplementary Materials

The following are available online at https://www.mdpi.com/article/10.3390/ijms222313069/s1.
