# Supplementary material for: Sustainable Stabilizer-Free Nanoparticle Formulations of Valsartan Using Eudragit® RLPO
Source: Int J Mol Sci. 2021 Dec 2;22(23):13069. doi: 10.3390/ijms222313069 (PMC8657980; doi:10.3390/ijms222313069)
Supplement: Supplementary file 1 [file ijms-22-13069-s001.zip › ijms-1490322-supplementary.pdf]

Supplementary Material

for

Sustainable Stabilizer-Free Nanoparticle Formulations of Valsartan Using Eudragit® RLPO

Eszter Hajba-Horváth, Andrea Fodor-Kardos, Nishant Shah, Matthias G. Wacker and Tivadar Feczkó

Table S1. Experimental parameters of valsartan-loaded Eudragit RLPO nanoparticles prepared by single emulsion-solvent evaporation method, the measured average particle sizes, polydispersity indexes, and the calculated encapsulation efficiency and yield. In the case of encapsulation efficiency the standard deviation of three parallel measurements are shown as well.

Sample ID	Eudragit RLPO quantity (mg)	Valsartan quantity (mg)	PVA conc. (%)	Mean size by intensity (%)	PDI	Encaps. efficiency (%)	SD	Yield (%)
**EUV28**	80.0	40.0	0.0	139.1	0.132	96.4	1.1	79.5
**EUV36**	80.0	40.0	0.25	169.7	0.120	96.4	0.9	82.9
**EUV20**	80.0	40.0	0.5	166.4	0.116	95.2	0.2	81.7
**EUV18**	80.0	40.0	1.0	162.3	0.090	94.2	2.7	78.0
**EUV26**	100.0	40.0	0.0	134.0	0.138	96.2	1.5	83.4
**EUV25**	100.0	40.0	0.25	160.7	0.137	91.8	1.2	86.1
**EUV37**	100.0	40.0	0.5	160.0	0.112	92.0	0.6	78.3
**EUV38**	100.0	40.0	1.0	159.6	0.109	92.3	2.9	76.8
**EUV27**	120.0	40.0	0.0	137.4	0.157	92.3	3.3	77.4
**EUV34**	120.0	40.0	0.25	146.7	0.122	91.6	1.4	71.1
**EUV24**	120.0	40.0	0.5	156.1	0.122	87.5	0.7	72.9
**EUV35**	120.0	40.0	1.0	149.9	0.111	89.8	0.	75.1
**EUV33**	160.0	40.0	0.	120.4	0.177	82.4	1.33	57.6
**EUV39**	160.0	40.0	0.25	139.7	0.158	84.6	1.2	60.9
**EUV40**	160.0	40.0	0.50	145.7	0.152	80.7	3.2	61.5
**EUV41**	160.0	40.0	1.0	148.4	0.155	77.5	1.2	59.2

Table S2. Experimental parameters of valsartan-loaded Eudragit RLPO nanoparticles examined in in vitro dissolution studies, the measured average particle sizes and polydispersity indexes.

Sample ID	Eudragit RLPO quantity (mg)	valsartan quantity (mg)	PVA conc. (%)	Mean size by intensity (%)	PDI
**EUV58**	160.0	40.0	0.0	134.4	0.174
**EUV59**	160.0	40.0	0.0	136.2	0.177
**EUV60**	160.0	40.0	0.0	136.7	0.195
**EUV61**	160.0	40.0	0.0	132.3	0.180
**EUV65**	160.0	40.0	0.0	133.9	0.199
**EUV67**	160.0	40.0	0.0	129.9	0.181
**EUV51**	160.0	40.0	1.0	150.2	0.164
**EUV52**	160.0	40.0	1.0	149.2	0.135
**EUV53**	160.0	40.0	1.0	154.6	0.175
**EUV54**	160.0	40.0	1.0	155.3	0.156
**EUV55**	160.0	40.0	1.0	153.1	0.157
**EUV66**	160.0	40.0	1.0	155.2	0.184
